# Graphene nanoparticles as data generating digital materials in industry 4.0

**DOI:** 10.1038/s41598-023-31672-y

**Published:** 2023-03-27

**Authors:** Muhammad A. Ali, Muhammad S. Irfan, Tayyab Khan, Muhammad Y. Khalid, Rehan Umer

**Affiliations:** 1 Department of Aerospace Engineering, Khalifa University of Science and Technology (KUST), Abu Dhabi, United Arab Emirates; 2 Present Address: Department of Aerospace Engineering, Khalifa University of Science and Technology (KUST), Abu Dhabi, United Arab Emirates

**Keywords:** Engineering, Materials science, Nanoscience and technology

## Abstract

One of the potential applications of 2D materials is to enhance multi-functionality of structures and components used in aerospace, automotive, civil and defense industries. These multi-functional attributes include sensing, energy storage, EMI shielding and property enhancement. In this article, we have explored the potential of using graphene and its variants as data generating sensory elements in Industry 4.0. We have presented a complete roadmap to cover three emerging technologies i.e. advance materials, artificial intelligence and block-chain technology. The utility of 2D materials such as graphene nanoparticles is yet to be explored as an interface for digitalization of a modern smart factory i.e. “factory-of-the-future”. In this article, we have explored how 2D material enhanced composites can act as an interface between physical and cyber spaces. An overview of employing graphene-based smart embedded sensors at various stages of composites manufacturing processes and their application in real-time structural health monitoring is presented. The technical challenges associated with interfacing graphene-based sensing networks with digital space are discussed. Additionally, an overview of the integration of associated tools such as artificial intelligence, machine learning and block-chain technology with graphene-based devices and structures is also presented.

## Introduction

An industrial revolution is a period of time in which significant changes occur in the way goods are produced to the extent that fundamentally transform the society, and is characterized by the introduction of disruptive technologies and novel production methods^1–3^. This typically leads to increased efficiency, reduced cost, greater production, and widespread economic and social impact^3^. The first industrial revolution (Industry 1.0) was characterized by the introduction of mechanical production methods using water and steam power^3,4^. Industry 2.0 saw the introduction of mass production using electricity and the assembly line^5,6^. Industry 3.0 introduced the use of information technology, computers and automation in production, leading to increased efficiency and customization^6^. Industry 4.0 takes this further by incorporating smart and autonomous systems, artificial intelligence, robotics, Internet of Things (IoT), cloud computing, and the integration of physical and virtual systems that leads to a further level of automation and data exchange^7–10^. Industry 4.0 is expected to gradually evolve into Industry 5.0 which will be characterized by further advancements in the above mentioned technologies^10–12^.

In Industry 4.0, the interconnection of the physical and virtual space is a crucial step which is necessary for realizing smart operations in the material design and manufacturing processes^13–16^. The physical space in a smart manufacturing setup refers to the manufacturing tools, raw materials and human resources. Whereas, the virtual space includes computational resources equipped with data storage and sharing capabilities as well as data analytics tools. The convergence of these two spaces is currently achieved through an array of embedded sensors or via imaging devices. However, these methods are inefficient and involve embedding of foreign objects within the material or structure. Replacing such devices with the material itself will revolutionize the paradigm of digital manufacturing. Such a material can be “smart” and capable of sensing and relaying the collected information or data to the virtual space in real-time.

Graphene and other 2D materials can act as the required interface and make the material directly communicate with the digital world^17,18^. Graphene and related 2D materials have been the focus of intensive research and development for over a decade, however products utilizing these materials have not captured the market yet. Graphene, termed as a “wonder material”, was anticipated to have wide range of applications ranging from electronics, civil/mechanical structures and water filtration to wearable technology, biosensors and medicine^19^. However, due to the scale and cost of production, these expectations could not be realized after more than a decade^20^. Currently, data generating devices (such as sensors) based on 2D materials are mostly in their initial Technology Readiness Levels (TRL). Further research is required in order to increase the technology readiness levels and have more sophisticated prototype systems manufactured for commercial deployment. In order to accelerate the path towards industrialization of 2D materials and increase their potential for future impact at commercial level, associated tools such as artificial intelligence and block-chain technology need to be developed and integrated with these devices. One of the potential applications of graphene nanoparticles is to impart multi-functionality to structures. These multi-functional attributes include sensing, energy storage, EMI shielding and property enhancement etc.^21–24^.

Graphene offers a number of fundamentally superior qualities that makes it a promising material for a wide range of applications, particularly in electronic devices. Graphene comes in a myriad of forms, such as nanoflakes, nanoplatelets, nanosheets, quantum dots, graphene oxide, graphite oxide, reduced graphene oxide, etc.^25^, with different forms provide different functionalities^22,26,27^. The form of graphene for a particular application may not be useful for other applications. For example, graphene used for EMI shielding cannot be used as a biosensor or as a transistor. Similarly, graphene used for energy storage is different from the one used for mechanical deformation sensing. The form of graphene focused here is the reduced Graphene Oxide (rGO) which is mainly used for sensing based on mechanical deformations. The versatility of graphene-based devices goes beyond conventional transistor circuits and includes flexible and transparent electronics, optoelectronics, piezo-resistive sensors, electromechanical systems, and energy storage devices^28^. Reduced Graphene Oxide based sensing has gained traction in the field of polymer composites very recently^29^. Graphene flakes and rGO can be embedded within a structure, such as a composite structure by either mixing it in the resin system or by coating on the fiber reinforcements^29^. The working principle of rGO based piezo-resistive sensors involves nanomaterials forming an electrically conductive network, and electrical tunneling between particles that is altered by external stimuli, resulting in changes in electrical resistance of the percolated network of graphene.

**Figure 1:**
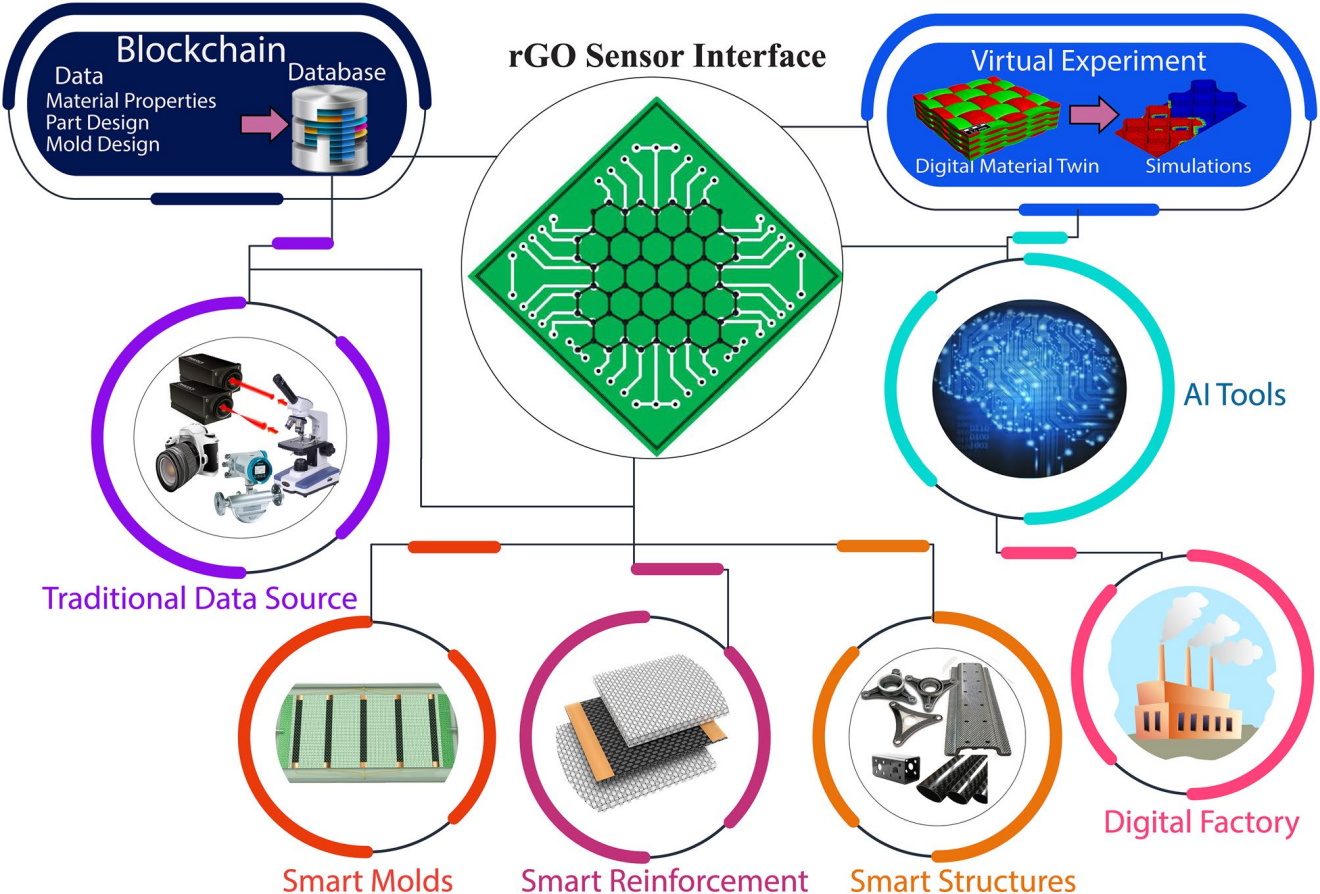
Flow chart illustrating the digitalization of composite structures using graphene nanoparticles as interface for creating a digital factory environment. Reduced Graphene Oxide based sensors along with traditional sensors can be incorporated in the manufacturing setup for digital manufacturing. Using the advanced tools such as block-chain technology, artificial intelligence, virtual simulations and digital twins, smart manufacturing can be achieved within Industry 4.0 framework.

The potential role of rGO sensors as an interface between the physical and cyber worlds in a digital manufacturing of fiber reinforced polymer composites is illustrated graphically in Fig. 1. Additional sensors such as pressure transducers, digital imaging devices, etc. could also be used to augment the information obtained via rGO based sensors. On top of the sensing system, a signal processing unit with diagnostic capabilities, and a data management system are also required for smart operations^30^. The block-chain technology is a promising tool for data collection and management, whereas artificial intelligence tools can provide the required signal processing capabilities. Hence, rGO based sensors, AI-powered tools and block-chain technology can form a triad that could enable smart manufacturing. Moreover, the database can be diversified with the help of simulation tools and digital twins.

In this article, we have reconnoitered the prospective utilization of graphene nanoparticles as digital materials within the context of Industry 4.0. First, we have explained how to use rGO as an embedded sensor, followed by the types of data generated by these sensors during the manufacturing process as well as during the service life of a structure. The use of block-chain technology and artificial intelligence tools for collecting and processing the data, and the role of digital twins in smart manufacturing is presented. The data generated using traditional and rGO based sensors can be collected and stored in an efficient and secure manner using block-chain technology. Machine and deep learning tools can be used for creating calibration, detection and predictive models using this database, which can analyze real-time signals captured using graphene-based sensors. In summary, we have presented a roadmap to converge three emerging technologies i.e. advanced 2D materials, artificial intelligence and block-chain in order to realize smart manufacturing in Industry 4.0.

## Creating reduced graphene oxide strain sensors

To make rGO based strain sensors for composites, graphene is used as a precursor, usually synthesized either by a top-down method or by a bottom-up approach^31^. The top-down approaches, such as mechanical exfoliation, oxidation–reduction of GO, liquid phase exfoliation and arc discharge, involve the structural breakdown of precursor such as graphite, followed by the interlayer separation to produce graphene sheets^32^. Chemical vapor deposition (CVD), epitaxial growth and total organic synthesis utilizing carbon source gas to synthesize graphene on a substrate, are examples of bottom-up technique^31^. Graphene nanoparticles and similar 2D materials can be embedded within a fiber reinforced composite structure either by dispersing them in the matrix or by coating them directly on the fiber reinforcements^33,34^.

### Reduced graphene oxide mixed within the matrix

In this approach, the polymeric resin (matrix) is modified by dispersing graphene nanoparticles within the resin, resulting in a traditional nanocomposite^35,36^. Enormous amount of useful data is gathered such as, mixing ratios, mechanical stirring force, centrifugal mixing parameters, etc. The data gathered during mixing of graphene nanoparticles in the resin system is useful to predict physical state of reduced graphene oxide, such as exfoliation and quality of reduction achieved, which can directly influence properties such as electrical conductivity, EMI shielding and a number of different mechanical properties^29^. However, the modified resin may also cause issues such as altering resin viscosity, particle agglomeration, premature gelation, filtering effect within the fabric while infusing the resin, and uneven distribution of the filler throughout the composite laminate^37,38^. These issues have hindered the practical application of rGO loaded resins and their composites, especially during the manufacturing of large and thick parts e.g. wind turbine blades, where mold filling can become very challenging.

### Reduced graphene oxide coating on reinforcements

Coating rGO directly onto the fibrous reinforcements instead of modifying the matrix is an alternative approach to overcome the issues highlighted above. In addition to imparting sensing abilities, the coating of reinforcements with rGO also provides the possibility of improving the mechanical and physical properties of the composite; hence, endowing multifunctional properties to the final structure^39^. Techniques for the depositing rGO onto fibrous reinforcements include, (i) chemical vapor deposition (CVD)^40^, (ii) electrophoretic deposition^41^, (iii) solution and spray coating^42^, and (iv) sizing containing rGO directly applied to the fibers during the fiber manufacturing process^43^. When deposited on the reinforcements, the composite part/structure becomes electrically conductive due to the formation of a network of meso-scale rGO nanoparticles^30^. When subjected to external stimuli, such as fluid pressure or mechanical forces, the conductive path is disrupted and the overall electrical resistance/conductivity of the part/structure is altered. This change in resistance/conductivity is measured and correlated with the external stimuli.

The overall resistance of the conductive network formed by rGO can be divided into three types: (i) intrinsic resistance of rGO, (ii) contact resistance, and (iii) tunneling/hopping resistance. This can be expressed using the following equation^29^

1 $$R=\sum_{i=1}^{N}{R}_{i}+\sum_{i=1}^{N}{fR}_{c}+\sum_{i=1}^{N}f{^{\prime}}{R}_{t}$$

where *R*_*i*_ is intrinsic resistance, *R*_*c*_ is the contact resistance and *R*_*t*_ is tunneling resistance. The key requirement for these sensors is the ability to detect any small changes in the overall resistance (ΔR). The signal is normally manipulated as a relative or Fractional Change in Resistance (FCR) rather than absolute measurements. The measured value is taken relative to a reference value (R_0_) and normalized by the same (R_0_), given as;

2 $$\mathrm{FCR}=\frac{R-{R}_{0}}{{R}_{0}}$$

where, R is the measured value and R_0_ is a reference value. The coated rGO can make the fabric material “digitally responsive” by generating signals which can be measured using any data acquisition (DAQ) system. The physical changes happening during manufacturing can easily be monitored, such as compaction response of the reinforcement, mold-clamping forces, resin pressure distribution, flow front tracking and resin cure kinetics, which were traditionally collected using external sensors and actuators that were not part of the material itself^44^. For process monitoring, the changes in electrical resistance can be expressed in terms of gain factor, which is a measure of the percentage change in the initial resistance of the structure. Apart from the signal, different parameters also need to be archived such as, sensor calibration, coating parameters, etc.^45^. There is huge amount of quantifiable parameters that can be recorded from the coating stage such as, concentration of the coating solution, sonication parameters (time, temperature and frequency), number of coating layers, rGO reduction time and temperature etc. These parameters affect the final resistance value, and hence the sensitivity of the rGO-based sensors^29^. The gathered data can be stored and analyzed for designing molds, selecting optimum injection gates and vents, measuring reinforcement permeability and predicting resin curing^46–48^.

## Data generated by reduced graphene oxide strain sensors

**Figure 2:**
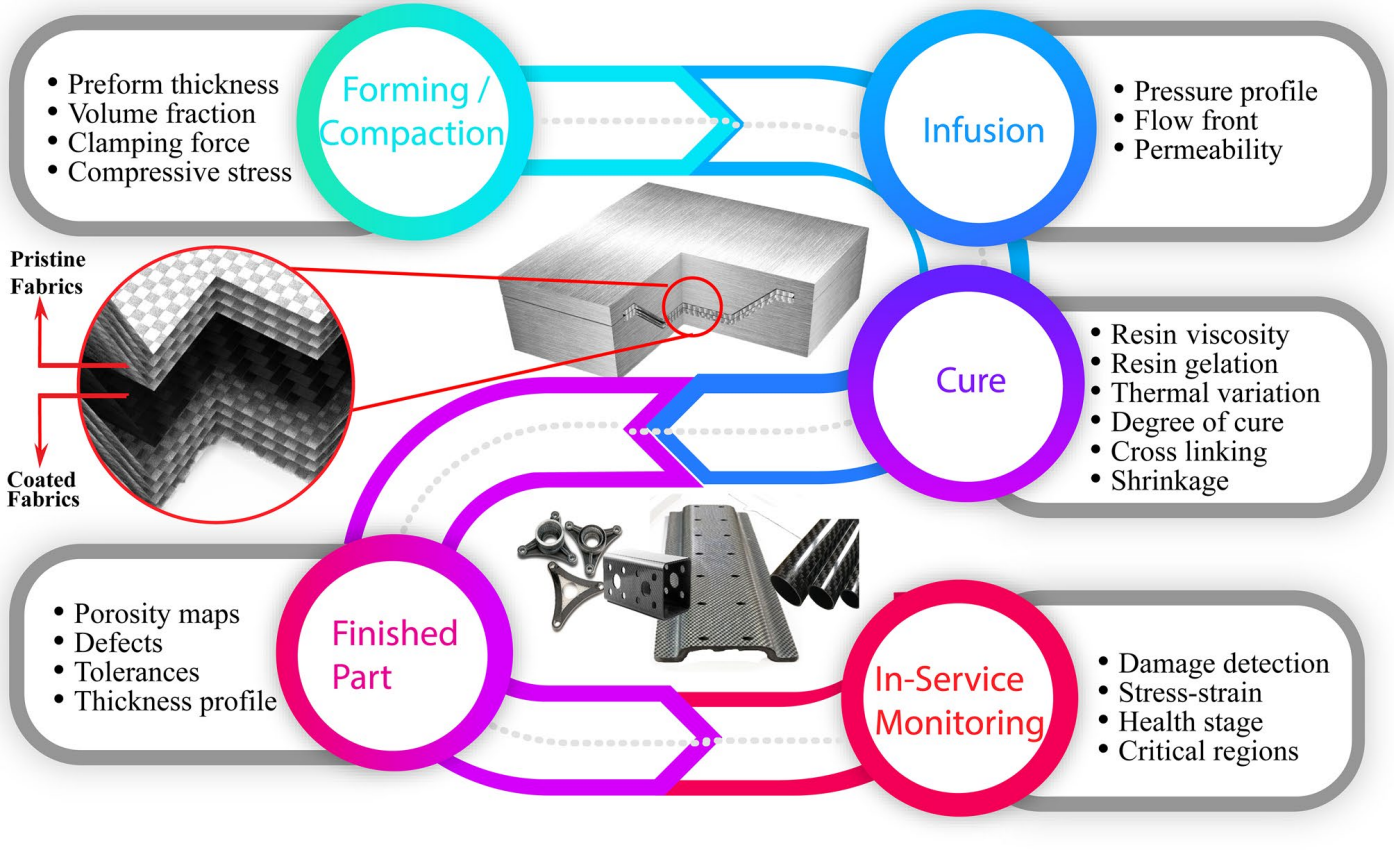
Data generated during the life cycle of a smart composite component, during and after manufacturing. Fabric compressibility is quantified by the applied stress required to achieve the target fiber volume fraction. The evolution of reinforcement permeability and flow characteristics are the important characteristics during the resin infusion followed by the cure kinetics of the resin. The distribution of stress within a structure is crucial for monitoring its health and for adopting prognostic measures. All aspects being monitored using in-situ coated fabrics.

While in operation, the rGO sensors generate signals that corresponds to various physical phenomenon/activities depending on the environment the smart material is exposed to. In a typical composites manufacturing process such as, Liquid Composite Molding (LCM) process, there are three main stages, i.e. compaction of the dry reinforcement, resin injection and resin curing, as shown in Fig. 2. All three stages are prone to process variabilities and need to be monitored using strain and pressure sensors. In the reported literature, rGO embedded fabric sensors have been employed for monitoring LCM processes^44,45^, which are some of the commonly used out-of-autoclave composite manufacturing processes. The rGO coated fabric based sensors are now being used in a variety of geometric forms (point sensors, line sensors, or area sensors) and configurations for monitoring applications^45^. It is also desirable that the concept of embedded sensors is applied to other composites manufacturing processes, such as filament winding and pultrusion for civil and construction industry. The embedded rGO-based sensors provide useful data at each stage throughout the manufacturing cycle, with vital information extracted related to the void content and structural health of the manufactured structure.

### Data generation during manufacturing

The first step in manufacturing of composites via Liquid Composite Molding (LCM) is the preforming step, in which the dry reinforcements are subjected to transverse compaction, so that they can conform to the mold shape and achieve the target part-thickness and fiber volume content. The compaction stage varies depending on the type of LCM technique used. The Resin Transfer Molding (RTM) is a closed-mold process where rigid mold platens apply high compaction forces on the reinforcements using a press, whereas, in Vacuum Assisted RTM (VARTM), the vacuum force is applied to compress the vacuum bag against the reinforcement laid on a single sided mold. During the compaction phase, uneven compression within a mold may result in thickness variations, particularly in the case of VARTM. In both cases, the applied compaction forces determine the fiber volume fraction of the composite, which in turn, determines the quality of the final part and mechanical properties of the composite. The rGO-based embedded sensors have been used to monitor compaction forces acting on the reinforcements in both VARTM and RTM processes. The rGO-based sensors are able to detect compaction forces of dry and resin impregnated reinforcements in the form of resistance change. During this stage, the mold clamping forces and stress relaxation data are required, which are usually proactively determined through characterization experiments^49^. These information are now being obtained in-situ via sensors based on 2D materials^50^. Recently Ali et al.^50^ have demonstrated that even a very complex time dependent phenomenon such as stress relaxation of the reinforcements in a closed mold can also be monitored using rGO and MXenes based embedded sensors.

During resin injection, the pressure distribution within the mold changes rapidly. This phenomenon is generally monitored using point sensors drilled within the mold^51–55^. rGO coated fabrics can act as an attractive alternative to these sensor arrays^44^. The resistance change data generated from the coated fabrics depends on resin conductivity and dielectric properties of the resin system used^44^. The conductivity/resistivity of graphene nanoparticles plays an important role when resin impregnates through the coated fibers. The gradual change in pressure inside the mold is also an indicator of resin impregnation captured via change in resistance of the embedded sensors. Moreover, race tracking and dry spot formation within the part can be detected by comparing signals from sensors placed at different spatial positions within the preform^45^. The interaction of resin with the sensors can provide information about the distribution of resin within the mold. It is also possible to make 2D plots of resin infusion process by spatial mapping during impregnation process^56,57^. This requires a virtual array of sensors, multiplexing system in combination with a Source Measure Unit (SMU) or similar resistance measuring unit.

The resistance of the embedded sensors is sensitive to gelation and crosslinking, as the resin observes shrinkage during these stages and apply compaction forces on graphene nanoparticles, thus resulting in a change in the overall electrical resistance^56^. Various stages of curing including, initial gelation, hardening (where resin shrinkage takes place) and post-cure are detected by monitoring relative changes in electrical resistance of the sensors such as described by Khan et al^45^.

### Data generation during post-manufacturing

Composite structures are frequently subjected to a number of loading scenarios in multiple applications throughout their service life. Depending on the type of application, these loads can range from high-velocity to low-velocity impact producing large deflections^58,59^. Any structural health monitoring system consists of sensing elements, preferably embedded within the structure and connected to a signal processing unit with diagnostic algorithms, and a data management resource^30^. Carbon based nanomaterials coated sensors have shown great potential in recent years for sensing applications in composite structures. Compared to carbon nanotubes, rGO and graphene flake sensors standout in their sensing applications due to higher aspect ratio and cost-effectiveness^60^. rGO embedded composite structures can be used to sense strain and damage during their lifetime. The mechanism of piezo-resistive sensing in FRPCs depends on whether rGO is coated on fabrics or mixed within the resin. When rGO is present in the matrix, an irreversible increase of the electrical resistance^61^ can be detected due to the initiation of cracks in the matrix and delamination of fabric layers. Alternatively, in the cases where rGO is coated directly on reinforcements, the conductive networks are confined to fiber surface, hence, detecting matrix cracks becomes relatively difficult. Nonetheless, enormous amount of data that is generated can be used for preventive arrangements of composite structures before any catastrophic failure happens^62^.

A lot of work has been reported on Structural Health Monitoring (SHM) where, composites were tested in different modes including tensile, compression, bending, impact, creep and stress relaxation^63^. A comprehensive literature review on the subject shows that a number of studies have reported successful implication of rGO-coated fabric sensors for monitoring the flexural response of composite structures. It is quite interesting to note that apart from precise strain sensing capability under flexural loading, these smart sensors can also exhibit a distinct response for tensile and compressive loads, if placed above and below the neutral surfaces in flexural loading^41,64^. A number of researchers have pushed these rGO coated sensors one step further to investigate their feasibility for sensing the repetitive long-term loading in composite structures. Remarkable repeatability in the piezoresistive response has been reported in both flexural and tensile cyclic loading for as high as 3000 loading cycles^65,66^. It is also worth mentioning that graphene nanoparticles based fiber sensors have also been adopted in complex composite structures for successful in-situ SHM. In fact, these smart sensors were yet again capable of reporting distinctive response to compression and tension loading based on their placement above and below the neutral surface^67^. Interestingly, a couple of studies recently further extended the use of rGO-coated sensors in the form of smart composite face sheets in honeycomb sandwich structures for in-situ SHM. Smart aerospace sandwich structures were not only sensitive to span length and core thickness^68^, but also exhibited distinctive responses to multiple loading rates in beams of any arbitrary width of interest^69^. Considering that sandwich composites based on Nomex™ honeycomb cores are an integral part of the modern aerostructures, these recent findings show remarkable potential in terms of sensing capabilities of active rGO-coated piezoresistive sensors in the aerospace industry.

Significant progress has been made thus far in terms of sensing the conventional mechanical response in composite structures. However, it is critically important to note that the inherited viscoelastic nature of the polymer resin and fiber reinforcements makes their mechanical response time-dependent, hence, the piezoresistive response of these smart sensors also becomes a function of time^70^. Therefore, it is very crucial to investigate the long-term creep and viscoelastic stress relaxation response using rGO-based smart sensors. Despite the importance of such response in long-term application of composite structures, this area of research has not been exploited properly yet. Irfan et al.^65^ conducted first of this kind of study based on rGO-based smart sensors, to investigate the effect of temperature on the mechanical performance of composites using dynamic mechanical analysis. The results were also compared with the response of MXene-coated fabric sensors under similar dynamic mechanical analysis using temperature sweep experiments. The results were quite promising as the sensors were not only capable to detect the thermomechanical response, but also detected the glass transition phenomenon and transition from glassy to rubbery region. In fact, rGO-based sensors exhibited smoother response compared to MXene-based sensors.

Therefore, rGO-based sensors have shown great potential for self-sensing applications in multiple industrial applications of composite structures. Nonetheless, self-sensing smart composite structures can be regarded as an emerging field, despite a number of limitations and challenges for researchers working in this field. Before their implication on an industrial scale, a number of areas need rigorous research. Some of these areas may include: (i) the scalability of these sensors; (ii) calibration; (iii) effect of other external stimuli, such as environmental factors; (iv) comparison with well-established conventional sensors for these applications, such as Piezoelectric Sensors (PZT) and Fiber Bragg Grating (FBG) sensors and (v) making these sensors smart enough to convey signal directly on portable devices such as mobile phones.

## The meta-verse of composites manufacturing

Given the fact that rGO-based sensors have great potential to be used in an industrial environment, their integration with cyber world is still a challenge and not much work has been done. In this section, we present a roadmap of Industry 4.0 technologies and how these technologies can use data generated through these sensors (as described in above mentioned sections) to create smart factories. A smart factory is self-adapting, and highly automated manufacturing environment capable of autonomously running entire production processes and making data-driven decisions^71,72^. Such a manufacturing setup has the ability to self-optimize performance and improve efficiency, flexibility, and quality control by self-adapting to new conditions through learning in real- or near-real time^73^. It integrates digital and physical systems through an interconnected network of machines, communication mechanisms, and computing power, and uses advanced technologies such as block-chain, artificial intelligence, machine learning etc., to gather and analyze data^74,75^. This integration is achieved through network of sensors and actuators enabling a physical system to access the capabilities of the virtual space or the “meta-verse”^75,76^.

Data gathered by rGO-based sensors, can be used for conducting virtual experiments and decision making in a smart factory. The rGO-coated sensors can feed digital information from the physical space to the digital space such as rGO mixing ratios, mold clamping forces, pressure distribution in the mold etc. This digital information comes in various formats (numeric data, images, time-dependent data, etc.). The role of digital space or the “meta-verse” is to collect this data securely, interpret the data and generate actionable commands. These actionable commands could be a decision tree that can enable/disable resin feeding lines based on the information gathered from the mold using rGO-based sensors.

### Creating digital material twins using data from rGO sensors

The concept of “meta-verse” is very broad and its key components are virtual/augmented reality using digital twins, artificial intelligence, block-chain, IoT etc. Digital Twin (DT) is one of the core components of Industry 4.0, which is termed as virtual replica or digital prototype of the physical process, fully integrated with the physical system and capable of performing virtual simulations in real time^77–79^. The virtual simulation is a key aspect of DT that requires continuous iterations between physical and virtual entities^80,81^. These simulations include physics based computational approaches (FEM/CFD)^82–87^ as well as data driven stochastics simulations^88–90^. The advantages of digital simulations over experimental procedures is evident in material consumption, labor hours and overall cost reduction. Apart from these advantages, these simulations can be used to generate datasets to be used in training and creating machine learning models. Although, such simulations cannot be performed in real-time, machine learning models based on the synthetic data can be useful^91^. The capabilities of digital twin are sometimes enhanced with the Virtual and Augmented Reality technologies^92^ that enable human–machine interactions^93,94^. For example, Perez et al.^95^ presented and validated a VR-enhanced DT for designing the automated process of a multi-robot manufacturing setup as well as its enhanced implementation and *in-operando* monitoring.

Digital twins are implemented at different yet interlinked levels^13^. In the context of composite structures, these levels include the design, manufacturing/assembly and in-services/operation phases^80^. At the design level, it is also known as “digital material twin” (DMT) which refers to the realistic computational models of the composite material that can be used for design verification and predicting the mechanical properties of the final composite, as well as estimating the process parameters such as the compaction response and resin flow properties within the reinforcing fibers^96–98^. These parameters are well captured by rGO coated fabrics (as described in previous sections) and this information can be stored and used to create “near to reality” DMTs. Moreover, rGO coated fabric sensors can also be used for the experimental validation of DT simulations.

Digital material twins for virtual manufacturing can be generated from different 3D scanning techniques, such as X-ray computed tomography (XCT)^99–103^. During production, DT is implemented at the shop-floor level for effective process monitoring, control and optimization^16,104,105^. Seon et al. created a DT for optimizing the de-bulking process of autoclave composites for mitigating void formation^106^. Zambal et al.^107^ generated DT for the detection of defects during carbon fiber layup using data collected from various sensors along with analytical modeling and finite element simulations. Finally, in the operational phase, DT is used for prognostics and diagnostics activities^108^. Milanoski et al.^109,110^ developed an FEM based DT for the structural health monitoring of stiffened composite panels by estimating the load acting on the structure using strain data acquired from Fiber Bragg Grating (FBG) sensors. Sisson et al.^111^ pursued a digital twin approach to optimize rotorcraft flight parameters by minimizing stress on critical mechanical components and through probabilistic diagnosis, prognosis, and optimization. Using the data collected from strain sensors, it is not only possible to detect the presence of the damage but also the evolution of the damage, hence remaining useful life of the part can also be predicted^109^. The knowledge about the health of the structures and parts helps in taking pre-emptive measures such as part replacement, repairing the damage, arresting cracks, etc.

### AI assisted digital manufacturing using data from rGO sensors

Artificial Intelligence (AI) generally refers machines that are designed to perform tasks that typically require human-like intelligence, such as perception, reasoning, and decision making^112–114^. Inherently, AI systems consist of data-driven mathematical models for inference and solving problems autonomously^114^. AI encompasses sub-fields of machine and deep learning, computer vision, natural language processing and cognitive computing, each of which focuses on different aspects of AI technology. Artificial intelligence and 2D materials are two of the disruptive technologies that are intertwined^115–117^. On one hand, the 2D materials could be an enabler for constructing devices for AI, such as memristors, photodetectors, etc.^118–122^. On the other hand, AI tools such as machine and deep learning can not only accelerate the discovery, design and optimization of 2D materials^123–126^, but also can interpret the signals generated by sensors based on 2D materials. Here, since we are discussing graphene as potential sensor, we will restrict our discussions to AI tools for signal processing.

The role of AI techniques in digital manufacturing using rGO sensors can be primarily viewed as a signal processing tool. Monitoring the manufacturing process usually involves detecting anomalies and measuring physical quantities such as pressure, temperature etc., which can be easily captured using rGO sensors. The real-time processing of signals with very low computational power makes these tools very attractive^127,128^. The signals measured by rGO-based sensors would normally be in the form of resistance/voltage/current measurements. These signals need to be converted to physical parameters such as pressure, stress, strain, temperature etc. through different calibration and correlation models^61,129–132^. Such calibration models can be easily developed using machine learning tools^17,50,133^. Zhu et al.^17^ employed a machine learning tool (principle component analysis) to predict the concentration of hydrogen gas from the measured response of rGO based gas sensor. Ali et al.^50^ calibrated MXene coated glass fabric sensors using supervised machine learning algorithms to correlate the compressive stress with the measured signal. Hajizadegan et al.^133^ extracted the concentration levels of the bio-chemical dopants from the harmonic spectrum of graphene-based harmonic sensors using artificial neural networks (ANN).

Other than the calibration models, AI tools can be easily employed for detection, inspection and monitoring tasks^134^. These tasks may include detection of resin race-tracking in molds^135^, flow disturbances^136^, and unfilled zones formation^137^ during the filling stage of an LCM process as well as inspection of broken-filaments during fiber production^138^. Novel AI-based methods for the inspection of the Automated Fiber Placement (AFP) process have also been presented by several researchers^139–143^. As part of health monitoring of structures, machine/deep learning models have been used for defect/damage detection^144–150^, characterization of cracks/delamination^151–153^ and classification of impact levels^154^. Yu et al.^154^ demonstrated that probabilistic Bayesian and traditional artificial neural networks can successfully classify the energy levels of different impact events based on the signals obtained from a network of piezoelectric sensors. Deep learning tools are particularly capable of such tasks when the signal is in the form of 2D/3D fields and maps^56,57^. In such cases, these models are not only able to detect these defects, but also locate them^152^.

Finally, the machine/deep learning-based surrogate/predictive models can be used for process simulations^155–157^ as well as for failure predictions in diagnostic and prognostic maintenance^158–160^. Using the data provided by a set of pressure sensors, Zhu et al.^161^ implemented a neural network model for the prediction of flow-front patterns at any impregnation time. Similar predictive models were also presented for forecasting resin cure^162^ and flow front progression^163^. Stieber et al. presented neural network based models FlowFrontNet^164^ and PermeabilityNets^165^ for the prediction of dry spot formation and permeability maps from a sequence of flow front images respectively. Pratim et al.^166^ presented an ANN framework to predict the life (durability) and residual strength (damage tolerance) of fiber-reinforced polymer (FRP) composites from real-time acquired dielectric permittivity of the material. Hassan et al.^167^ used genetic algorithms for failure prediction in self-sensing nanocomposites based on conductivity changes observed via electrical impedance tomography.

**Figure 3:**
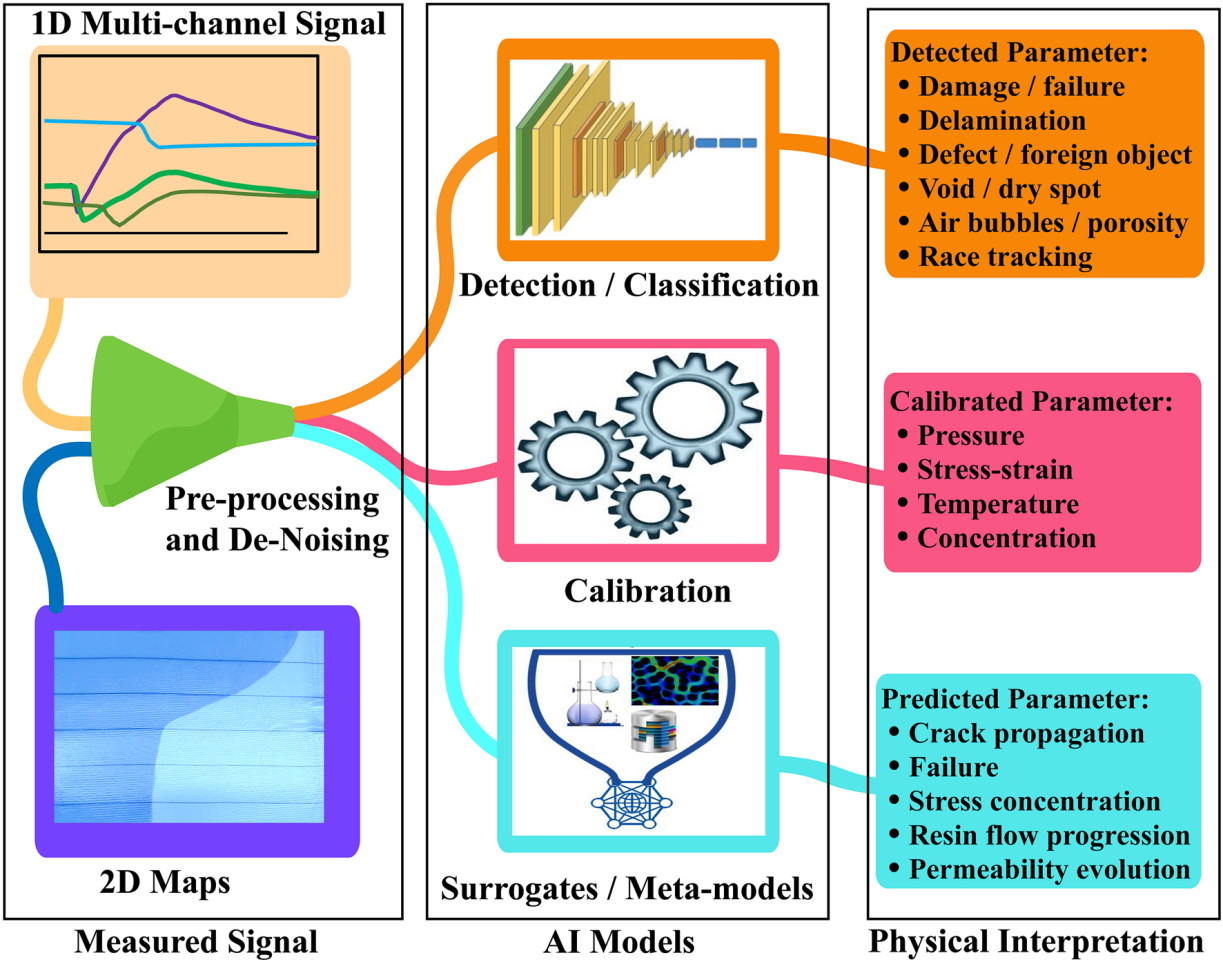
The use of machine and deep learning models for various tasks in composites manufacturing in Industry 4.0. The common application of such models include calibration of the sensors, anomaly detection by analyzing the signals and perform predictive tasks in real-time.

In summary, these tools can be integrated within the digital manufacturing setup as calibration, detection and predictive models as summarized in Fig. 3. Moreover, these models can be periodically re-trained as availability of new data without losing the old weights, hence, truly updating the whole manufacturing process. Some of the models discussed here used data generated from traditional sensors or synthetic data rather than data collected by piezoresistive rGO sensors. However, the methods discussed here can easily be adapted for analyzing data obtained via rGO sensors.

### Block-chain technology based on rGO sensor data

As AI tools can analyze the data collected through rGO-based sensors efficiently, the block-chain technology can collect and manage data in a secure, trustworthy and traceable manner^168,169^. By definition, a block-chain is an evolutionary list of immutable records, called blocks, which are linked together using cryptography and stored on a decentralized network of computers or nodes in chronological order^170^. Block-chain technology employs a self-executing piece-of-code, known as smart contracts, to automate the process in a much reliable and trustful way^171^. Currently, this technology is being exploited extensively by the financial and banking sectors, healthcare and supply chain sectors^172^. For example, the block-chain technology can be employed in the supply chain of fiber reinforced composite materials, in particular, in handling temperature-controlled transportation, handling and storage of prepregs on a tamper proof distributed ledger^173^.

**Figure 4:**
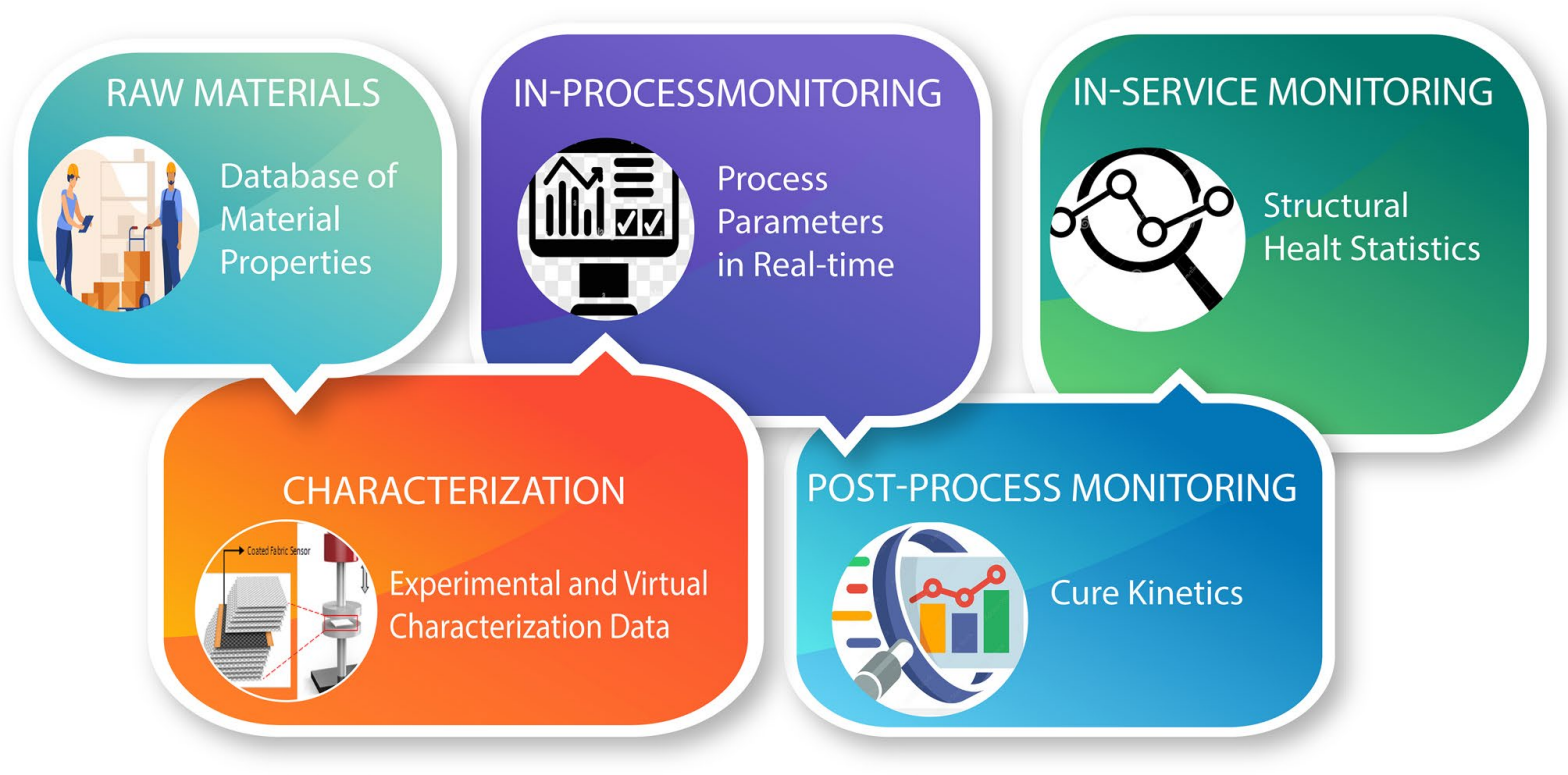
Data collection at various stages of composites manufacturing using block-chain technology. The data generated at various manufacturing stages including the data sheets of the raw materials and in-service signals can be gathered in an efficient and secure manner by using block-chain technology.

While using rGO as a sensing element for the manufacturing of fiber reinforced polymer composites, the data is generated at various stages, which includes physical properties as well as process parameters. These stages form the multi-echelon supply chain that comprises raw materials, manufacturing process and the finished components/structures^173,174^. The nature and format of data varies depending on the processing step, and includes numeric values, time/temperature dependent curves, or even two/three dimensional fields, as well as subjective descriptions. All the data generated at each step can be collected and stored in an efficient and secured manner using block-chain. A conceptual illustration of the use of block-chain in collecting and storing the generated data is illustrated in Fig. 4. Apart from the data directly collected from rGO sensors, the data related to physical characteristics of the reinforcement and matrix, as well as data generated from physical and virtual experiments is also crucial for efficient processing. The physical characteristics of the reinforcement and matrix are usually provided by the supplier (first block in Fig. 4). These properties are then validated as well as new characteristics are determined via characterization experiments and virtual simulations using digital twins (second block in Fig. 4). The shape of the part to be produced, which will be in the form of a 3D geometry, is another important piece of data. Mold designs and other process parameters depends on the type of manufacturing method used. In case of LCM, the process parameters include the number and location of inlet/outlet ports, injection pressure, etc. For processing prepregs, the cure cycle and temperature are the main parameters. The rGO coated materials can play a vital role in in-situ data acquisition during the process. The inspection of finished parts will produce more data related to the quality of the part, such as porosity maps and void content and tolerance levels^175^. Finally, while in service, the smart structure based on rGO sensors will generate signals related to its structural health, which can be managed in the maintenance log-book on the block-chain ledger^176^. Apart from direct involvement, block-chain can also help in creating DT’s^169,177^ and work in conjunction with artificial intelligence to have an overall impact^178^. Nevertheless, block-chain technology is a secure, large-scale and reliable data collection and management tool for implementing smart operations using networks of sensory elements^179^, including rGO based sensors.

## Concluding remarks and outlook

There are numerous challenges and opportunities for the technological applications and market penetration of graphene nanoparticles as a digital materials in various real-world applications. It is vital to consider these challenges prior to the largescale commercialization of graphene as a sensing element in fiber reinforced polymer composites, and to make them compatible with the standards of Industry 4.0. The material selection process is of paramount importance as there are several 2D materials now available, and the chosen 2D material will affect not only the processing steps but also the final sensing properties of the product. The economy of scale is also a factor when choosing a 2D material. Atomistic modeling can be a tool to narrow down the material selection for a particular application. This becomes very important when multifunctional composites are involved. The engineered 2D materials such as MXenes can be designed to obtain optimized properties. Atomistic modeling can also help in making hybrids of two or more materials. Synthesis of good quality materials is also a challenge, especially if the processes are not well defined in literature and practice. It must be decided if in-house synthesis is required or off-the-shelf options may work for an application.

Adding graphene and other 2D materials into the process chain is the next challenge. There are numerous ways in which graphene can be incorporated into composites, for example, mixing in the resin system, coating on the reinforcements, weaving a coated tow into the reinforcement fabric, or coating the final composite with the graphene solution. There is no single solution, the user needs to decide which method is optimum for the target application.

Sensor manufacturing is another closely related challenge. It is also important to decide about the size, number and spatial location of sensors in a structure. Embedding a sensor in a complex 3D shape while maintaining its sensing properties can be a difficult task. It is also important to keep in mind the manufacturing process, where it would require different approaches to embed a sensor. Whichever technique is used to embed graphene nanoparticles into the composite, it is important to quantify the sensing capability of the composite to sense any physical changes.

Sensor calibration is a major challenge in this field, especially when inter-lab sensors are involved. There is no standardization of these sensors yet, however for a commercial application, a standardization protocol is desirable. The property retention of sensors over time is also a critical factor. Environmental factors such as temperature and humidity might affect the sensing capability over time. This is also important in commercial sensors such as FBG sensors, and a routine inspection is performed to ensure the working of these sensors in real-world applications such as bridges. In the same way, graphene nanoparticles based sensors should have a provision for inspection over time. Meanwhile, in lab environments, accelerated tests can be performed to quantify the property retention.

A large-scale production system is essential for the commercialization of graphene nanoparticles as a viable digital material. As mentioned earlier, various commercial vendors are available for provision of graphene materials however, the application of graphene in different fields poses unique challenges. Graphene and other 2D materials are viable nanomaterials to be used as smart sensors in fiber reinforced composites. They can provide process and structural health monitoring at every stage of composites manufacturing and application. In addition, these materials can also enhance other base properties of the neat composite, including mechanical properties and EMI shielding.

The entities in meta-verse are far more mature than 2D materials. The digital space has seen tremendous advancements in computational capabilities that include cloud computing, big data analytics, IoT and artificial intelligence (AI). However, their integration with sensors based on 2D materials have not been achieved yet. Even, compatibility of various digital tools is also not clear. One of the key characteristic of the block-chain technology is publically available information. However, most of the information in a manufacturing environment are of propriety nature. In this regard, consortium or federated block-chains can be used where the information is restricted to a target audience only. The AI tools are data driven, and require carefully curated data sets for training. Such type of data is scarce at the moment but is expected to grow with time. Lastly, the concept of digital twin based on graphene nanoparticles sensors is also in its conceptual phase. The growth of all these technologies together can bring in the true essence of Industry 4.0. There is no doubt that there are rich opportunities for the application of graphene and other 2D materials in this area. It is a high time that academics and composites industries including aerospace and automotive sectors should work together to solve challenges in the field and aim for the wide-scale adaptation of graphene as a digital material to reap the benefits of this wondrous material.

## Data Availability

All data generated or analyzed during this study are included in this published article.
